# Supplementary material for: Highly Efficient Bioconversion of trans-Resveratrol to δ-Viniferin Using Conditioned Medium of Grapevine Callus Suspension Cultures
Source: Int J Mol Sci. 2022 Apr 15;23(8):4403. doi: 10.3390/ijms23084403 (PMC9026456; doi:10.3390/ijms23084403)
Supplement: Supplementary file 1 [file ijms-23-04403-s001.zip › ijms-1661947-supplementary.pdf]

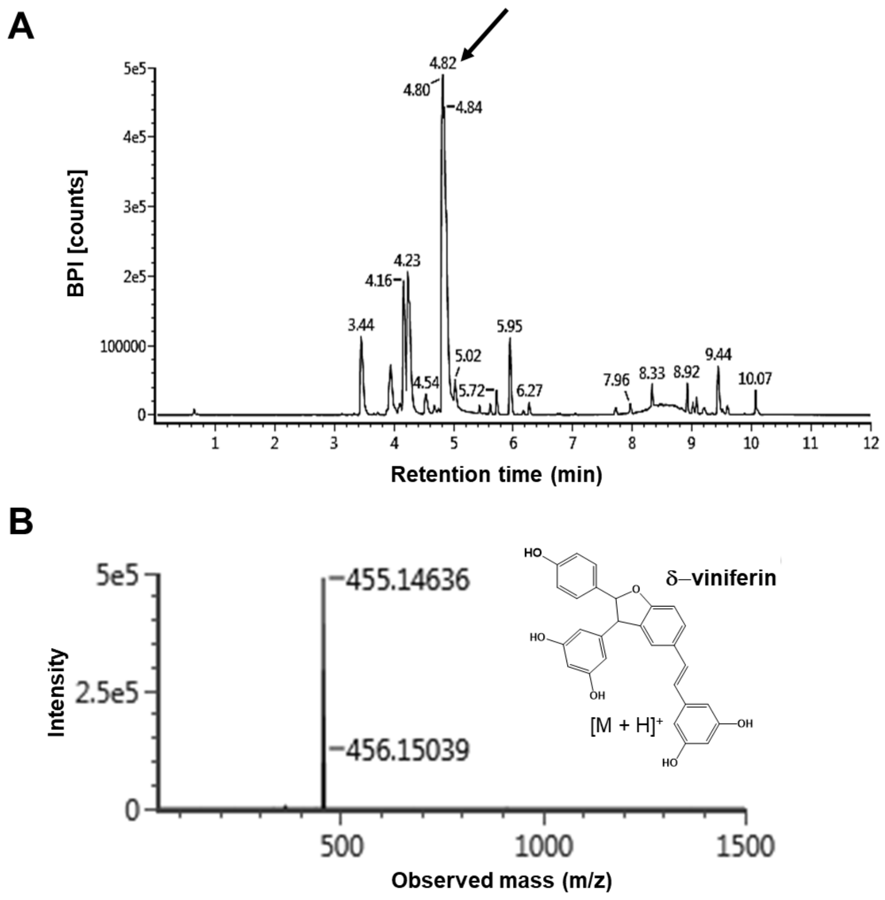

Figure S1. Representative UPLC-Q-TOF MS profile of the bioconverted δ-viniferin using conditioned medium in grapevine callus cultures. (A) Chromatogram of UPLC-Q-TOF of the bioconverted δ-viniferin. (B) Mass spectrum of bioconverted δ-viniferin analyzed by ESI-positive mode.

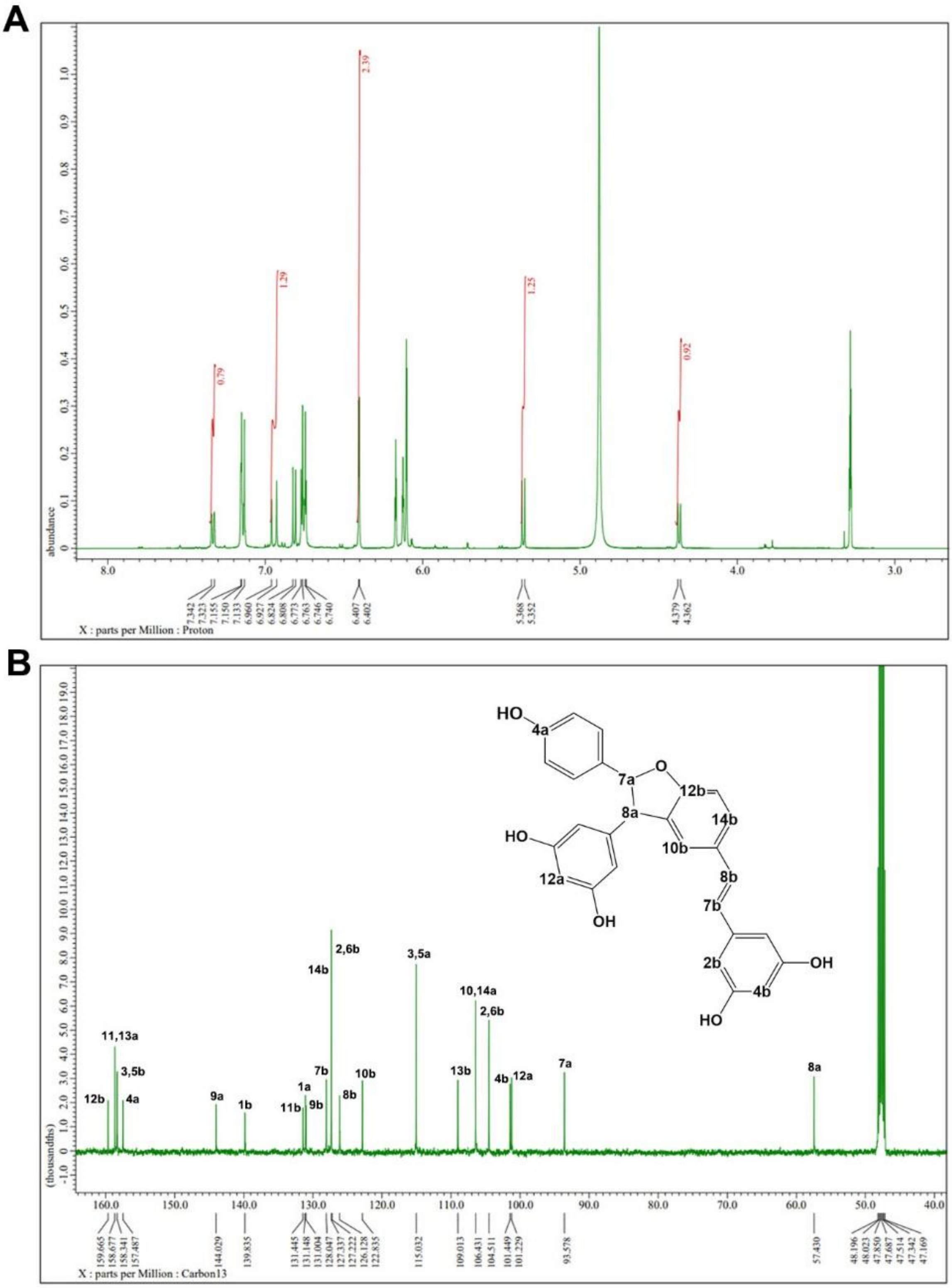

Figure S2. ^{1}H- and ^{13}C-NMR spectral data (methanol solution) of bioconverted δ-viniferin using conditioned medium in grapevine callus cultures. δ-viniferin: (A) ^{1}H-NMR (500 MHz, methanol-d_4); δ 7.33 (1H, dd, *J* = 9.5, 2.5 Hz, H-14b), 7.17 (1H, s, H-10b), 7.15 (2H, *d*, *J* = 9.0 Hz, H-2(6)a), 6.96 (1H, *d*, *J* = 16.5 Hz, H-7b), 6.82 (1H, *d*, *J* = 16.5 Hz, H-8b), 6.80 (1H, *d*, *J* = 8.0 Hz, H-13b), 6.75 (2H, *d*, *J* = 8.5 Hz, H-3(5)a), 6.40 (2H, *d*, *J* = 2.0 Hz, H-2(6)b), 6.18 (1H, t, *J* = 2.0 Hz, H-12a), 6.16 (1H, t, *J* = 2.0 Hz, H-4b), 6.15 (2H, *d*, *J* = 2.0 Hz, H-10(14)a), 5.35 (1H, *d*, *J* = 8.0 Hz, H-7a),

4.36 (1H, *d*, *J* = 8.5 Hz, H-8a); (B) ^{13}C-NMR (125 MHz, methanol- *d*$_4$); δ 159.6 (C-12b), 158.6 (C-11, 13a), 158.3 (C-3, 5b), 157.4 (C-4a), 144.0 (C-9a), 139.8 (C-1b), 131.4 (C-11b), 131.1 (C-1a), 131.0 (C-9b), 128.0 (C-7b), 127.3 (C-14b), 127.2 (C-2,6b), 126.1 (C-8b), 122.8 (C-10b), 115.0 (C-3, 5a), 109.0 (C-13b), 106.4 (C-10,14a), 104.5 (C-2,6b), 101.4 (C-4b), 101.2 (C-12a), 93.5 (C-7a), 57.4 (C-8a)

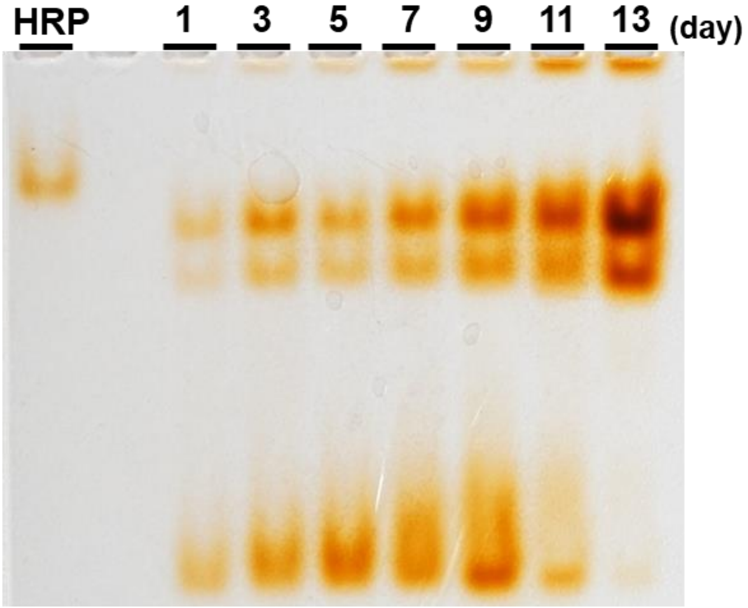

Figure S3. Guaiacol peroxidase zymography of grapevine CM from callus suspension cultures. The CM supernatants were harvested in two-day intervals for 13 days during callus suspension culturing. The non-boiled CM samples (30 μL) were dissolved in 5X sample buffer and electrophoresed through 12% native PAGE.
